# Vectored delivery of anti-SIV envelope targeting mAb via AAV8 protects rhesus macaques from repeated limiting dose intrarectal swarm SIVsmE660 challenge

**DOI:** 10.1371/journal.ppat.1007395

**Published:** 2018-12-05

**Authors:** Hugh C. Welles, Madeleine F. Jennewein, Rosemarie D. Mason, Sandeep Narpala, Lingshu Wang, Cheng Cheng, Yi Zhang, John-Paul Todd, Jeffrey D. Lifson, Alejandro B. Balazs, Galit Alter, Adrian B. McDermott, John R. Mascola, Mario Roederer

**Affiliations:** 1 Immunotechnology Section, Vaccine Research Center, National Institutes of Health, Bethesda, Maryland, United States of America; 2 Institute for Biomedical Sciences, The George Washington University, Washington, DC, United States of America; 3 Ragon Institute of MGH, MIT and Harvard, Cambridge, Massachusetts, United States of America; 4 AIDS and Cancer Virus Program, Leidos Biomedical Research, Inc., Frederick National Laboratory of Cancer Research, Frederick, Maryland, United States of America; King's College London, UNITED KINGDOM

## Abstract

Gene based delivery of immunoglobulins promises to safely and durably provide protective immunity to individuals at risk of acquiring infectious diseases such as HIV. We used a rhesus macaque animal model to optimize delivery of naturally-arising, autologous anti-SIV neutralizing antibodies expressed by Adeno-Associated Virus 8 (AAV8) vectors. Vectored transgene expression was confirmed by quantitation of target antibody abundance in serum and mucosal surfaces. We tested the expression achieved at varying doses and numbers of injections. Expression of the transgene reached a saturation at about 2 x 10^12^ AAV8 genome copies (gc) per needle-injection, a physical limitation that may not scale clinically into human trials. In contrast, expression increased proportionately with the number of injections. In terms of anti-drug immunity, anti-vector antibody responses were universally strong, while those directed against the natural transgene mAb were detected in only 20% of animals. An anti-transgene antibody response was invariably associated with loss of detectable plasma expression of the antibody. Despite having atypical glycosylation profiles, transgenes derived from AAV-directed muscle cell expression retained full functional activity, including mucosal accumulation, *in vitro* neutralization, and protection against repeated limiting dose SIVsmE660 swarm challenge. Our findings demonstrate feasibility of a gene therapy-based passive immunization strategy against infectious disease, and illustrate the potential for the nonhuman primate model to inform clinical AAV-based approaches to passive immunization.

## Author summary

Antibodies are the humoral component of an immune response against an invading pathogen or vaccine immunogen. For challenging vaccine targets, as an alternative to active vaccination to induce the immune system to generate antibodies, current research is exploring the delivery of these proteins to populations at high risk of infection as prophylactics against infectious diseases, like HIV, RSV, and Ebola, amongst others. Passive vaccination via purified protein will require periodic reinjection to retain protective levels in subjects, adding a barrier to large scale coverage. Alternatively, delivery of antibodies using gene therapy may provide a one-time passive vaccination alternative. This strategy comes with its own hurdles, including anti-vector immunity, anti-drug immunity, physical limitations of vector uptake and the need to confirm antibody functionality. To date, many passive vaccinations strategies remain untested in humans. Non-human primate models of infection are frequently useful for predicting the success of vaccine candidates or concepts. Here, we characterize and optimize a rhesus macaque model for the delivery of anti-viral antibodies via the gene therapy vector adeno-associated virus. Lastly, we demonstrate the ability of the mAbs to protect against viral challenge. Our work demonstrates the feasibility and utility of vectored delivery of antibody transgenes in rhesus macaques. We hope this model of antibody delivery may be applied to various disease models in non-human primates and will inform clinical trial design of passive vaccination against infectious diseases.

## Introduction

Antibodies mediate protection against infection for nearly all traditional licensed vaccines. To achieve protective antibody titers, active immunization has historically been employed. More recently, given advances in monoclonal antibody isolation and production technologies, delivery of prophylactic antibodies via passive vaccination is being considered for pathogens where active vaccination strategies have not succeeded. Advantages of passive immunization include better efficacy, enhanced coverage against multiple disease strains or serotypes, greater tolerability, and economics. Strategies to prevent infection via prophylactic administration of monoclonal antibodies are advancing clinically, with success against a variety of diseases (primarily in animal models): respiratory syncytial virus, Influenza A, hepatitis C, herpes simplex, Ebola, Rabies, cytomegalovirus, Hendravirus, and HIV.[1–3] As with all protein-based regimens, obstacles include cost of production[4,5], maintenance of functional drug levels[6–8], and rejection by anti-drug antibody responses.[7,9–11]

Genetic delivery of antibody via viral vectors such as adeno-associated viruses (AAV) is particularly exciting given preclinical and clinical studies demonstrating persistent expression of transgene drug products.[12–18] Gene therapy provides a potential one-time administration strategy, after which protein products are produced *in vivo*, and persistently accumulate to functional levels.[16–19] Adeno-associated virus (AAV) is a safe gene therapy vector capable of delivering transgenes long term.[20,21] The clinical success of AAV-vectored gene therapy for hemophilia highlights the potential to replace infusion of purified F.VIII or F.IX protein in humans with a single administration, resulting in millions of dollars saved in infusions of purified clotting factors.[12,14,22,23] Used appropriately, AAV vectored gene therapy has an excellent safety record, has been approved clinically as the drug Glybera, and has showed successes in human trials for gene replacement in multiple diseases (lipoprotein lipase deficiency, Leber’s congenital amaurosis, and aromatic L-amino acid decarboxylase deficiency).[21,24,25]

The use of AAV to deliver antibodies against infectious disease has been adopted by the field of HIV preclinical research and recently extended to human clinical trials. (NCT017455, NCT03374202) To date, two phase I clinical trials delivering anti-HIV envelope broadly neutralizing antibodies (bNAbs) via AAV have begun. However, the field of HIV is awash with candidate antibodies that may be useful for prophylaxis. To determine the best passive immunization candidates for HIV prevention via gene therapy, *in vivo* NHP models are the best animal model. To date, five studies have provided proof of principle that HIV/SIV envelope targeting antibodies can be delivered to NHP via AAV.[26–30]

Initial studies delivered non-simian antibodies, simianized antibodies, or antibody-like molecules showed high frequency of loss of expression due to anti-drug antibody (ADA) responses.[26–28,30,31] Chimeric models suffered from issues of compatibility between host immune system and the infecting virus or non-simian antibody. Specifically, the generation of ADA was reported to abrogate any protective effect of the delivered antiviral molecules. Here, we show that we can largely circumvent the challenges of anti-drug immune responses previously described by delivering autologous, naturally-arising mAbs, as would be done clinically. We demonstrate the feasibility of AAV8-based delivery of native anti-SIV Env-specific mAbs, show durable transgene antibody accumulation in serum and mucosal compartments with a low frequency of ADA responses in species-matched immunocompetent outbred hosts, and observed a protective effect of VIP against SIV challenge.

This platform has the potential for long term delivery of anti-viral immunity against SIV, an established model of natural infection for HIV.[32–35] Here we used AAV8 to deliver antibodies directed towards the CD4 binding site (CD4bs) and variable loop 1 (V1) region of its envelope. We established and optimized a platform of passive immunization to deliver species matched antibodies where transgene expression is durably sustained, anti-drug antibody generation is infrequent, and transgene mAbs translocate to mucosal compartments where they may act prophylactically. We describe the unique glycan profiles and maintenance of neutralization function of transgene mAbs. Lastly, we performed challenge studies to demonstrate the protective capacity of vectored immunoprophylaxis (VIP). Together, our data inform clinical trials testing the AAV platform to deliver HIV broadly-neutralizing antibodies to prevent infection.

## Results

### Anti-SIV mAb delivery via infusion

To test the feasibility of passive immunization of rhesus macaques using anti-SIV env mAbs, we performed an initial series of antibody infusions. Animals received purified ITS01-LS (n = 2) or ITS09.01-LS (n = 2) intravenously four times at 3 or 30 mg/kg at approximately 9 week intervals. The LS mutation is reported to enhance antibody persistence in human and macaques, and was chosen here to mirror clinical passive immunization.[36,37] For these doses, the serum antibody concentration demonstrated a typical two-compartment decay, as expected for infused antibodies (S1A Fig). Infused antibodies translocated to rectal mucosa as expected (S1B Fig). At peak post infusion, mucosal ITS01-LS represented up to 2% and 3% of total IgG in rectal biopsies and swabs, respectively. Likewise, mucosal ITS09.01-LS represented up to 2.3% and 1.2% of total IgG in rectal biopsies and swabs, respectively. The amount of mucosal mAb was correlated to the plasma levels (rectal Weck-Cel spears, R = 0.60, p ≤ 0.01; biopsies, R = 0.83, p ≤ 0.01) (S1C Fig). Antibodies demonstrated consistent half-lives of 24.7 ± 8.5 and 13.4 ± 3.6 days for ITS01-LS and ITS09.01-LS across 4 infusions, respectively (S1D Fig).

The consistent serum antibody decay rates suggest the absence of anti-drug antibody (ADA) responses, despite the introduction of the “LS” mutation to increase neonatal FcR binding and extend circulating half-life of mAbs.[36] We found no evidence of ADA by direct assay of sera, even after 4 successive infusions, save for one animal A03684 (S2 Fig). This animal tested positive for ADA against ITS01 and ITS09.01 at one and two timepoints, respectively. All three of these measurements were only slightly above the limit of detection for ADA. In this animal, despite receiving only infusions of ITS09.01-LS, the serum was reactive to both ITS01 and ITS09.01 Fab. Additionally, the half-life of ITS09.01 following the 2^nd^ and 4^th^ infusions did not decrease dramatically compared to others. Thus, this response was an extremely weak, cross-reactive ADA response.

Based on these data, we moved forward with testing AAV-vectored approaches to long-term delivery of mAbs in vivo. Given the vector availability at the time, antibody Fc in AAV constructs did not include the LS mutation.

### Down selection of AAV candidates

Candidates selected for NHP AAV studies were first tested for expression and neutralization in mice *in vivo* using AAV8. Mice were injected IM with 2.5 x 10^10^ gc/animal (n = 1/mAb). All antibodies were detected in sera at high concentration and exhibited binding to SIVmac239 gp140 by week 8 (S3A Fig). Transgene antibody neutralization capacity in serum was assayed to ensure proper function. ITS01, ITS06.02 and ITS11 demonstrated neutralization while ITS08 and ITS10 failed to neutralize (S3B Fig). ITS01 and ITS06.02 were selected for advancement to NHP *in vivo* testing based on these data to have one anti-CD4bs and one anti-V1 AAV construct. ITS11 was not selected given its poorer expression and lower potency than ITS06.02.

### Proof of concept

**Fig 1:**
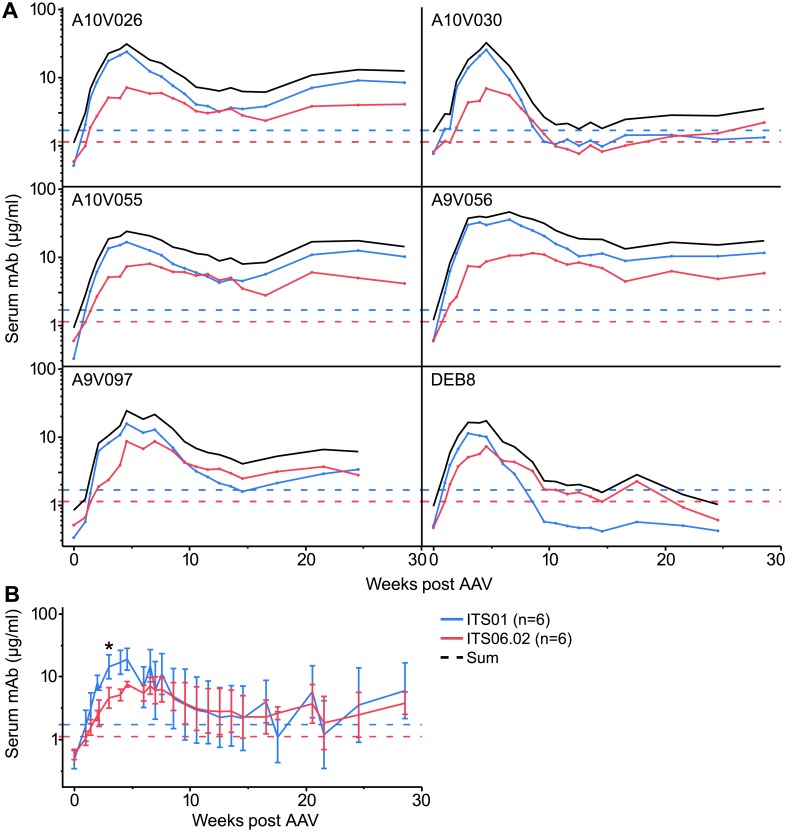
Stable transgene mAb expression following AAV8 administration in NHP. Rhesus macaques were injected with both AAV8-ITS01 and AAV8-ITS06.02 at 1 x 10^13^ gc/mAb/animal in the right and left quadriceps, respectively. (A) Serum ITS01 (blue lines) and ITS06.02 (red lines) and their sum (black lines) are shown individually for each animal over time following injection. (B) The average and standard deviation of expression levels are shown across all animals. Dashed lines indicate the lower limit of detection by binding to SIVmac239 gp140 (2 standard deviations above negative control sera) for ITS01 (blue), ITS06.02 (red). Asterisks denote statistically significant difference between mean ITS01 and ITS06.02 expression at week 4 (p ≤ 0.05).

To determine the utility of AAV to provide adequate and durable expression of anti-SIV env mAbs in NHP *in vivo*, we injected six rhesus macaques intramuscularly with 10^13^ gc/animal each of AAV8-ITS01 and AAV8-ITS06.02 separately, one in each quadriceps to avoid potential mismatched Ig heavy/light chain pairing. Detectable transgene mAb began at week 2, peaked at week 5 and came to setpoint around week 14 (Fig 1A). ITS01 and ITS06.02 expression peaked at 21.2 ± 8.6 μg/ml and 8.6 ± 1.7 μg/ml, respectively. At peak serum antibody levels (day 28 post AAV), ITS01 reached higher levels (mean ITS01 = 18.2 μg/ml; mean ITS06.02 = 5.2 μg/ml; p = 0.03; Matched Pairs Wilcoxon Signed Rank), but this difference disappeared by day 42 (Fig 1B). In two animals, serum abundance of both mAbs (DEB8) or ITS01 (A10V030) fell below the limit of detection at set point, which was later found to be due to ADA, discussed below.

### Anti-vector immunity

**Fig 2:**
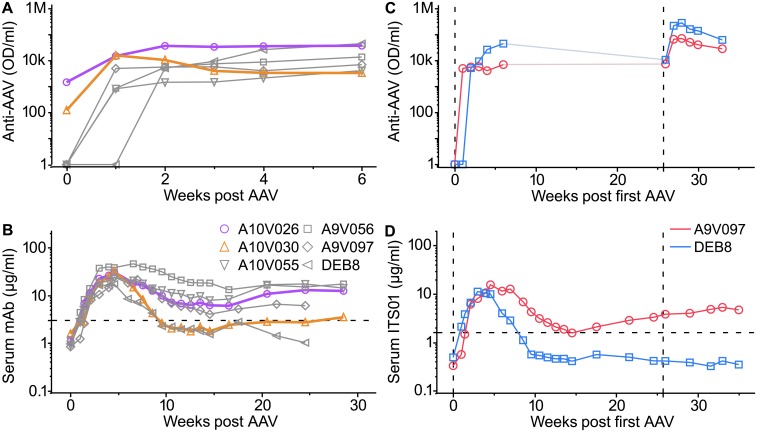
Impact of anti-vector immunity on transgene expression. (A) Anti-vector reactivity and (B) expression of ITS01 is shown for the six animals of Fig 1. Animals with low (A10V030; orange) and intermediate (A10V026; purple) pre-existing anti-vector immunity are highlighted. After 6 months, two of these animals were re-injected with 1 x 10^13^ gc AAV8-ITS01 in the right quadriceps. (C) Anti-AAV8 reactivity was boosted, however (D) no increase in serum ITS01 was observed. Anti-AAV8 measurements are in arbitrary optical density (OD_450nm_)/ml units.

Pre-existing anti-vector antibody might prevent the initial transduction. Two of six pilot animals were found to harbor low and intermediate pre-existing anti-vector serum reactivity (Fig 2A). Despite this immunity, both animals were successfully transduced and their serum mAb abundance fell within the range for all six monkeys (Fig 2B).

Notably, all animals developed high anti-vector immunity within 2 weeks of injection (Fig 2A). We chose one animal that maintained (A9V097) and one (DEB8) that lost detectable serum ITS01 to investigate the possibility for repeat AAV administration to boost serum mAb levels. Animals were injected with 10^13^ gc of AAV8-ITS01 each at 180 days after initial administration. Re-administration boosted the high anti-vector immunity approximately 10-fold higher within one week (Fig 2C), indicating a successful “take” of the injection. Nonetheless, serum ITS01 abundance did not change in the 10 weeks following the second administration (Fig 2D). Thus, high level anti-vector humoral immune responses will inhibit repeat transduction by the same vector.

### Dose and route optimization

**Fig 3:**
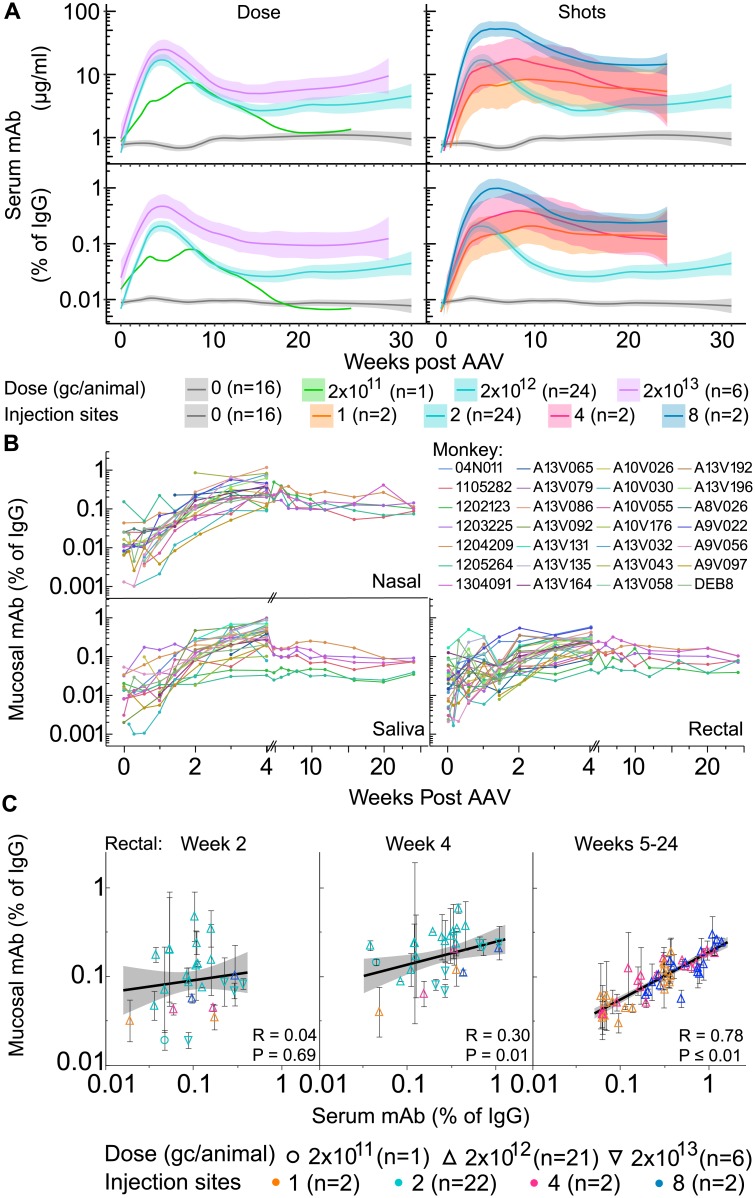
Dose and injection site number titration of AAV administration. (A) Absolute (upper panels) or fractional (lower panels) serum antibody levels of ITS01, ITS06.02 or both are shown as a smoothed fit with 95% confidence interval shading for varying doses (left panels) or shots (right panels). At the lowest dose, data for a single animal is shown (the other one animal showed no expression of transgene). Animals received 2 injections into the quadriceps with AAV8-ITS01 and/or AAV8-ITS06.02 at low, medium, high doses (2 x 10^11^ (n = 1; green), 2 x 10^12^ (n = 24; cyan), 2 x 10^13^ (n = 6; violet) gc/animal, respectively). Controls received no injections (n = 16; gray). For varying shots, animals received 2 x 10^12^ gc/animal spread across 1, 2, 4, 8 injection sites (1: R quadriceps (n = 2; orange), 2: both quadriceps (n = 24; cyan), 4: both quadriceps and gastrocnemii (n = 2; magenta), 8: both quadriceps, gastrocnemii, deltoids and biceps (n = 2; blue), respectively). (B) Fractional mucosal transgene IgG is shown for individual macaques in mucosal compartments (n = 28). A scale adjustment is an x-axis break to emphasize early mucosal accumulation denoted by diagonal hashes between 4 and 5 weeks post AAV. (C) The correlation between serum versus rectal transgene mAb is shown for weeks 2, 4, and 5–24 post AAV. Points are colored by number of shots and point shapes denote dose, low, medium or high as noted in the legend.

Given the successful delivery of antibody via AAV8, we reduced the dose by 10- and 100-fold to economize vector production. At medium dose (2 x 10^12^ gc/animal; n = 24), comparable expression was observed compared with high dose (2 x 10^13^ gc/animal; n = 6) (Fig 3A, S4A and S4E Fig). At the low dose (2 x 10^11^ gc/animal; n = 2), one of two animals showed no serum accumulation of mAb, while the other showed low peak and set point expression near baseline. Both animals demonstrated anti-AAV8 seroconversion. The animal lacking serum transgene accumulation was removed from further analyses to demonstrate correlations between dose and output levels. When comparing peak (weeks 4 post AAV) and set point (weeks 18–54 post AAV), serum transgene mAb, no differences among doses were observed that reached significance (p ≤ 0.05). The highest dose trended to mildly higher levels, but the increase was about two-fold despite a 10-fold higher dose. These data suggested a saturation of the site of injection occurring at or near the medium dose, which might be expected if the number of muscle cells transduced by needle injection is limited by physical parameters.

To further explore this limitation, we administered the same total medium dose divided across 1 (n = 2), 2 (n = 24), 4 (n = 2), or 8 (n = 2) injection sites at a single time point. Peak serum transgene mAb accumulation followed a near-linear dependence on the number of injection sites (Fig 3B, S4B and S4F Fig). However, these differences waned by week 24; nonetheless, the two animals receiving 8 injections exhibited a more consistent, high-level expression. These data suggest that optimization of plasma transgene mAb accumulation from AAV should be aimed at delivery devices that can target many distinct injection sites rather than increasing the dose of the vector.

To ensure that AAV transgene mAbs are translocated to mucosal surfaces, where they may act prophylactically against pathogens, we measured transgene mAb from nasal, rectal and buccal swabs. Assaying mAb abundance revealed long term accumulation at these mucosal sites (Fig 3B and S6A Fig). Translocation of transgene mAbs to rectal mucosa correlated strongly with relative serum mAb abundance at peak and late timepoints (week 4, R = 0.30, p = 0.01; weeks 5–24, R = 0.78, p ≤ 0.01) (Fig 3C). Different delivery modalities did not diminish mucosal accumulation (S5B Fig), and the correlation between mucosal and plasma antibody levels tended to improve over time as the mucosal antibody equilibrated (S6B Fig).

### Anti-drug antibody responses

**Fig 4:**
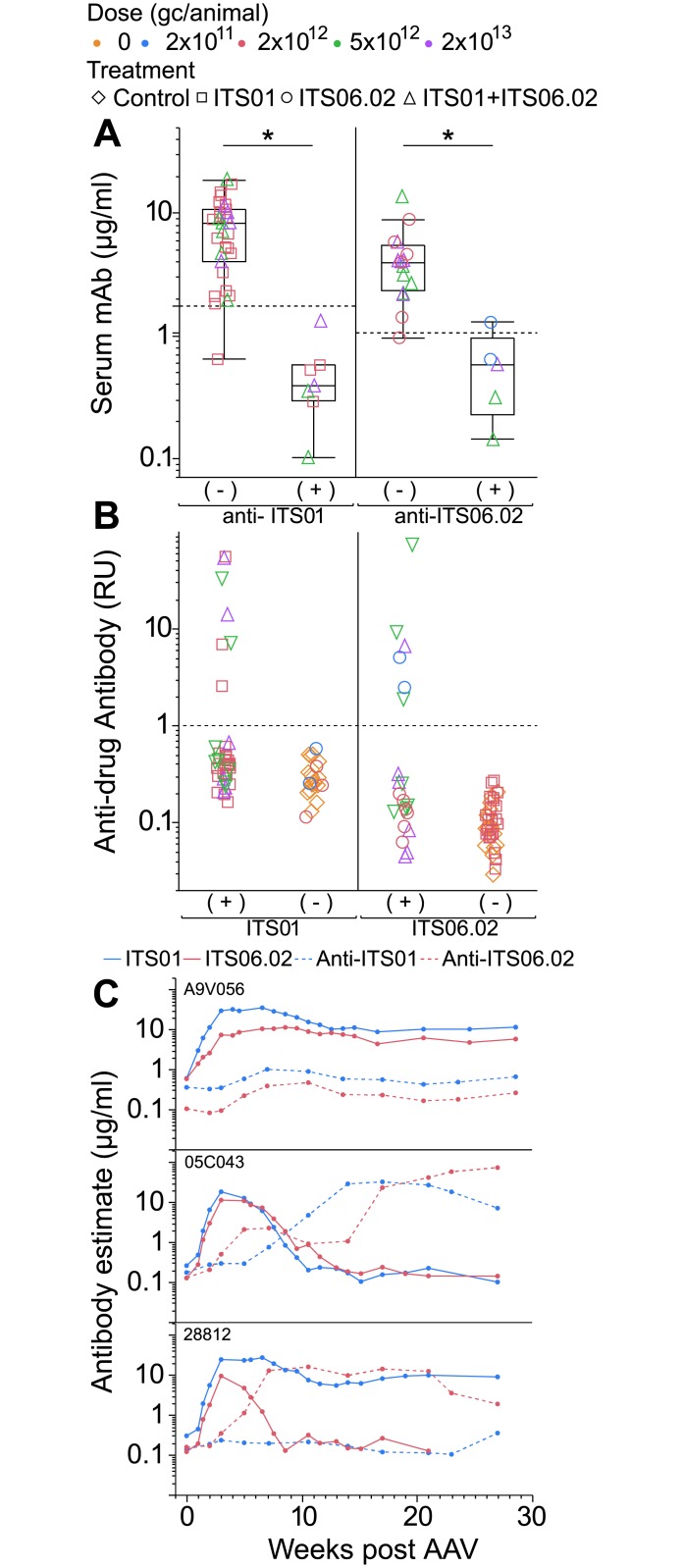
Infrequent ADA responses accompany loss of serum transgene mAb. (A) Serum mAb measurements in injected animals that did (+) or did not (-) develop anti-ITS01 (left) and/or anti-ITS06.02 (right). Hashed lines denote the limit of ITS01 or ITS06.02 positivity (2 SD above mean of negative control serum) at 1.3 μg/ml, blue and 0.8 μg/ml, red, respectively). Asterisks indicate statistical significance (p ≤ 0.05). (B) Polyclonal serum ADA responses were quantified in relative μg/ml equivalents to monoclonal control anti-idiotype antibodies. Hashed lines denote anti-ITS01 or anti-ITS06.02 threshold of positivity set at 1 RU. Dose and AAV treatments are coded by color (Control, low (2 x 10^11^ gc/animal; blue), medium (2 x 10^12^ gc/animal; red), intermediate (5 x 10^12^ gc/animal; green), and high (2 x 10^13^ gc/animal; purple)) and shape denotes antibody delivered: control (diamonds), ITS01 (squares), ITS06.02 (circles), ITS01 + ITS06.02 (upward triangles), ITS01 + ITS06.02 + ITS52 (downward triangles). (C) Serum ADA response estimates (hashed lines) and AAV mAb estimates (solid lines) are shown for ITS01 (blue) and ITS06.02 (red) for 3 representative examples of animals that developed no ADA (top panel), ADA against both mAbs (middle panel), or ADA against only one mAb (bottom panel).

Prior passive immunization attempts in rhesus macaques using mAbs derived from humans, antibody-like molecules or non-native anti-S(H)IV mAbs have been complicated by frequent and rapid anti-drug antibody generation.[26–29] We quantified serum anti-drug antibody responses in macaques receiving anti-SIV env mAb via AAV. At late timepoints, (9–31 weeks post AAV), serum mAb (ITS01 or ITS06.02) was below the limit of detection in all animals where ADA responses were generated (p ≤ 0.01) (Fig 4A). However, serum ADA responses were infrequent: approximately 20% of animals developed measurable ADA against the antibodies they received via AAV (7/38 anti-ITS01; 6/22 anti-ITS06.02). No cases were observed of cross reactive ADA: i.e. no animals receiving ITS01 generated anti-ITS06.02 antibodies or vice versa, suggesting these responses are specifically anti-idiotypic (Fig 4B).

To characterize the basis of the serum ADA response, a subset of animal sera, including from all animals that generated ADA at late timepoints, were assayed for ADA over time. Polyclonal serum ADA were quantified using a monoclonal ADA standard, and thus are quantified relative to monoclonal μg/ml equivalents. These estimates were overlaid with serum antibody expression levels for three representative cases, where no ADA (A9V056), ADA to both mAbs (05C043) or ADA to only one mAb is generated (28812) (Fig 4C). Typically, ADA responses arose approximately 8–12 weeks post AAV and persisted at high levels up to 180 days.

### Antibody glycosylation profiling

AAV8 predominantly transduces hepatocytes and muscle cells.[30,38] We compared the Fc glycosylation profile of transgene mAbs secreted by these cell types in the transduced animals to those of B/plamsa cells during infection or tissue culture-derived antibody. Specifically, we quantified Fc-glycan species of Env-specific mAbs from the sera of AAV transduced animals at week 4 post AAV, mAb infused animals (293 or CHO-produced) day 5 post infusion, and chronically SIV infected animals (polyclonal sera) via capillary electrophoresis. Because of the descriptive nature of this analysis comparing many glycan species, only those significant differences with p ≤ 0.001 by Steel-Dwass nonparametric comparisons are reported below.

**Fig 5:**
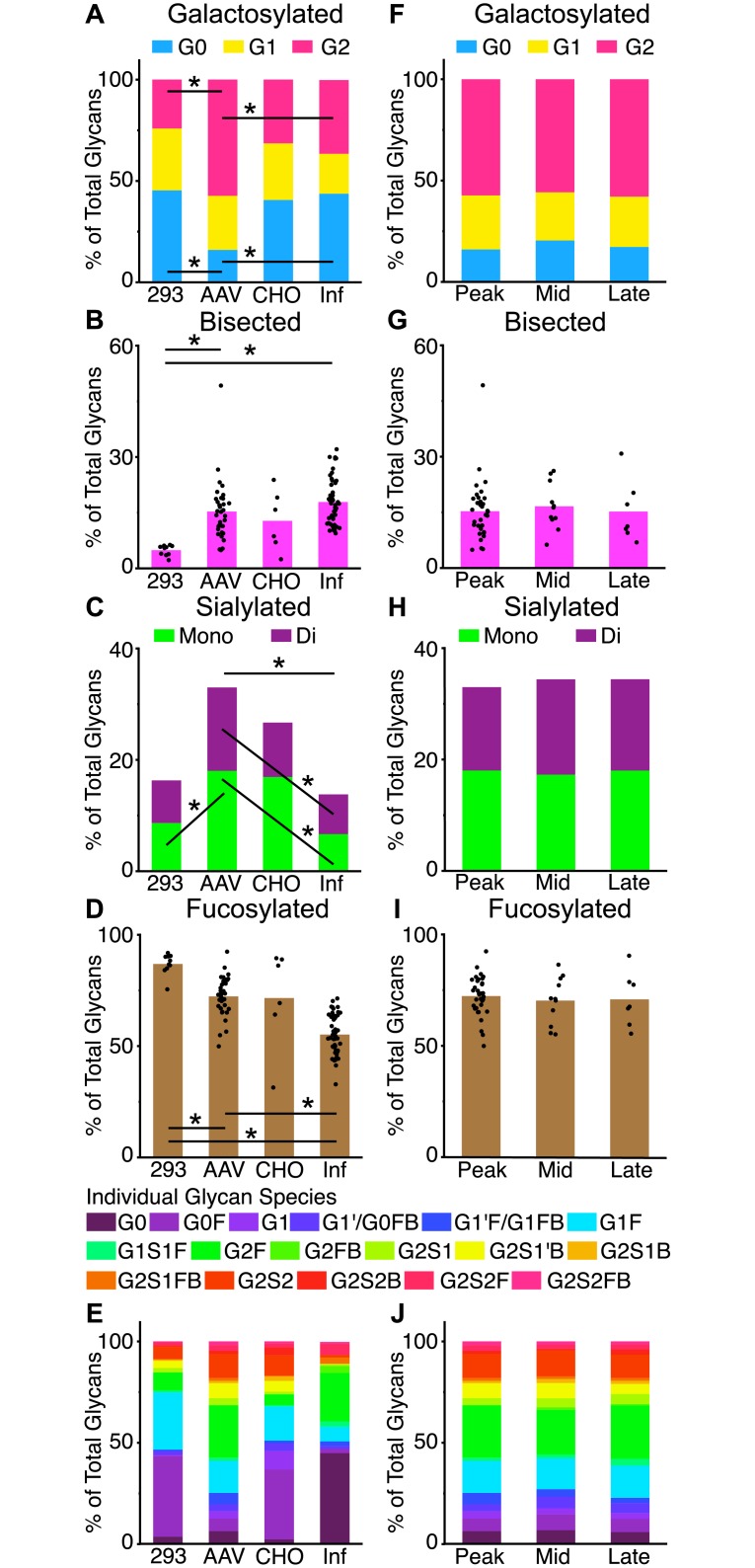
AAV-expressed mAb exhibit a distinct glycosylation profile from *in vitro* purified or naturally secreted mAb. Glycans were eluted from serum anti-SIV env reactive mAbs from passively immunized or SIV-infected animals and analyzed via capillary electrophoresis. (A-D) Analysis of total grouped glycan species are displayed as a percent of total glycans (E) Individual glycan species of are quantified as a percent of all glycans and compared by derivation. (F-I) Analysis of total grouped glycan species of AAV expressed NAbs are compared across time. (J) Individual glycan species of are quantified as a percent of all glycans and compared across time for AAV derived antibodies. (A-D, F-I) Differences between groups reaching p ≤ 0.001 are denoted by black lines. (E, J) Differences between groups of individual glycans reaching p ≤ 0.001 are described in text.

Bulk Fc-glycan analysis from antibodies revealed AAV transgene mAbs had more digalactosylated and fewer agalactosylated mAbs than 293 or infection derived mAb (Fig 5A and S8A Fig). Bisected glycans were lowest in 293 derived mAb compared to AAV and B/plasma cell derived mAb (Fig 5B and S8A Fig). Transgene mAbs and CHO produced cells were highly monosialylated compared to infection derived mAb, with AAV derived mAbs also being highly disialylated compared to infection derived mAb. AAV derived mAbs also bore more total sialylation than infection derived antibody (Fig 5C and S8A Fig). AAV derived mAb had less fucosylation than 293-produced mAb, but more than those derived from infection (Fig 5D and S8A Fig).

To further dissect these differences, the individual glycan species were compared. 293-derived mAbs contained much higher levels of G0F and G1F glycans than AAV and infection derived antibody (Fig 5E and S8B Fig). AAV derived mAb bore higher G1, G1’/G0FB, G1’F/G1FB, G2S1, and G2S1B Fc-glycans than all others. Infection derived antibody was enriched in G0, G1S1F, G2F, G2FB, G2S1FB and G2S2F and found to be lowest in G1, G2S1’B, G2S2 and G2S2B. Where cell culture derived mAb (293 and CHO) were equivalent, G1 and G2S1FB were enriched glycan species from these sources.

Hepatocytes have been shown to be highly permissible to AAV transduction and contribute to transgene serum expression. However, these cells are short lived compared to muscle cells and may contribute only temporarily to transgene expression. Thus, we compared the glycan profiles of transgene mAb secreted at 3–4 (peak), 8 (mid), and 12 (late) weeks post AAV. Bulk galactosylation, sialylation, fucosylation and biection did not change over time (Fig 5F–5I). The individual glycan profile showed no change over time for AAV transgene mAbs (Fig 5J). Individual glycan profiles for 293 and CHO derived mAbs also did not change over time, aside from the selective retention of 293 derived bisected mAb species (S7 Fig).

### *In vitro* antibody function

**Fig 6:**
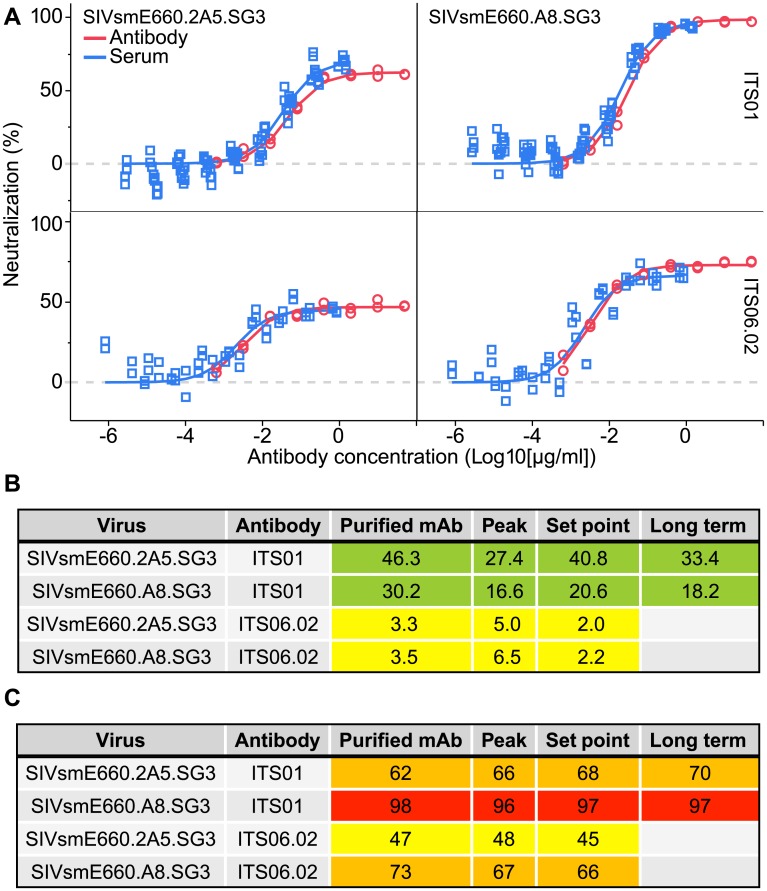
AAV-expressed serum mAb retains full neutralization activity. (A) Standard neutralization regression analyses are shown for ITS01 (upper) and ITS06.02 (lower) from purified mAb (red, open circles) or sera from AAV injected rhesus macaques (blue, open squares) (n = 6). Neutralization of SIVsmE660 clones 2A5 (left) and A8 (right) for mAbs and sera (ITS01, week 24; ITS06.02, weeks 5–8) are overlaid. (B) EC_50_ (ng/ml) and (C) maximum neutralization (%) are listed for ITS01 and ITS06.02 purified or from transduced NHP sera. Antibodies in serum are derived from AAV injected animals at peak (week 4), set point (weeks 5–8) or long term (week 24) post AAV. EC_50_ are displayed in ng/ml with the following color coding: 10–90 Light Green; 1–9 Yellow. Maximum neutralization values are displayed as percent with the following color coding: 20–49.9% Yellow; 50–74.9% Light Orange; 75–94.9% Orange; 95–100% Red.

Antibody glycosylation has been demonstrated to alter function, particularly, neutralization. Additionally, ADA responses can target antibody paratopes, blocking their ability to neutralize effectively. In our initial mouse screening for AAV administration, serum ITS08 and ITS10 no longer had neutralizing capability (S3B Fig), although the reasons for this loss of function were not explored. Sera from animals transduced with a single AAV transgene antibody were compared against purified mAb. When normalized for transgene antibody abundance, the serum neutralization regression curve aligned with that of purified cell culture produced mAb (Fig 6A). This concordance in EC_50_ was observed for all timepoints, for animals receiving either ITS01 and ITS06.02, and against both neutralization-sensitive and -resistant SIVsmE660 clones tested (Fig 6B). Similarly, the maximum neutralization capacity between transgene and purified cell culture derived mAb was consistent across all assays for ITS01 and ITS06.02 (Fig 6C). Thus, there was no change in the SIV-neutralization parameters of the mAbs expressed by the AAV transduction.

### Vectored immunoprophylaxis

**Fig 7:**
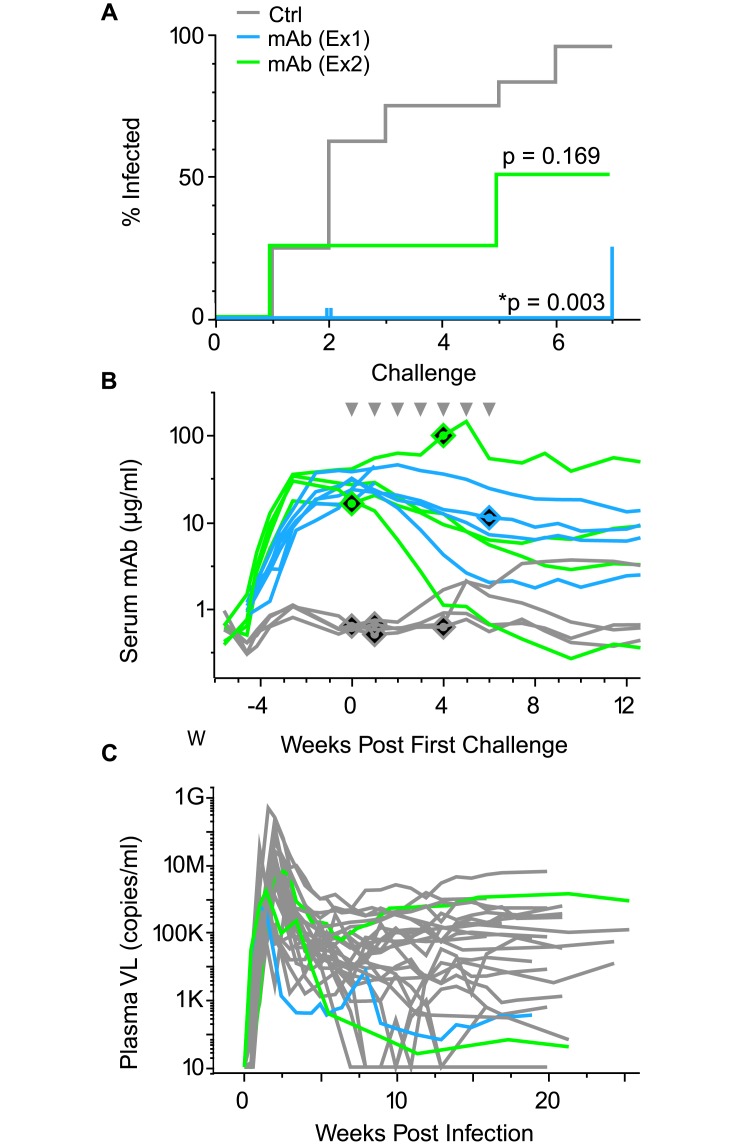
Vectored immunoprophylaxis mediates a protective effect against limiting dose SIVsmE660 swarm challenge in rhesus macaques. (A) Limiting dose intrarectal challenges were administered weekly as denoted in the Kaplan-Meier curve on the x-axis to animals receiving mAb (ITS01+ITS06.02) via AAV in our initial (blue, n = 6) and repeat (green, n = 4) groups or no mAb controls (gray, n = 24). Two animals were censored from analysis after challenge 2 due to false positive viral loads (vertical ticks) (B) Serum anti-SIV env transgene mAb was quantified and plotted (y-axis) versus time (x-axis) for mAb pretreated animals and four contemporaneous control animals. Diamonds indicate timepoint of infection corresponding to animals’ expression level line. Challenges at AID_0.33_ are denoted as grey downward facing triangles above. (C) Plasma viral loads (y-axis) are plotted versus time post infection (x-axis).

Delivery of antibodies via AAV has been proposed as a one shot alternative to repeated infusions for protection against infectious diseases. Given the translocation of mAbs expressed via AAV to mucosal surfaces and retention of function, we hypothesized that protection against repeated low dose administration of SIV could be observed. We enrolled Indian origin macaques that received ITS01 and ITS06.02 into a challenge regimen to test the hypothesis that vectored delivery of anti-viral antibodies could protect against SIV infection. We began challenges five weeks post AAV to allow for adequate accumulation of transgene mAb in plasma and at mucosal surfaces then performed seven limiting dose intrarectal challenges using SIVsmE660 swarm at AID_0.33_. The passively immunized group had a 3.1% rate of infection, with only a single infection in 32 challenges compared to the 34% rate of infection of historic controls (n = 20). VIP reduced the rate of infection by ~90% compared with historic controls (p = 0.003) (Fig 7A). Two monkeys were removed from the challenge regimen after two challenges due to false positive viral loads (later confirmed by retesting sera from the same time points). As a sensitivity analysis, we refit our models with these two animals excluded. The results were unchanged (p = 0.006). To confirm this protective effect, we repeated this challenge regimen with an AAV vectored mAb group (n = 4) and contemporaneous controls (n = 4). The mAb pretreatment showed a trend towards a similar level of protection versus contemporaneous controls (75%, p = 0.237), with 2 infections in 20 challenges. These infections occurred despite >10 μg/ml of serum transgene mAb in AAV treated animals (Fig 7B). All three infections in mAb pretreated animals resulted in productive infection (Fig 7C). These animals had a geometric mean peak viral load 0.67 logs lower than control animals. Combining data from all challenges with the same virus swarm stock, the AAV transduced animals (n = 10) showed a protective effect of 83% (p = 0.002) versus controls (n = 24).

## Discussion

Our study demonstrates the utility of NHP to model passive immunization delivering native anti-viral mAbs via infusion or AAV. Infused mAbs behave as expected for native antibodies and were detected at the mucosal surfaces within a short timeframe following infusion.[7,36,37,39] The successful translocation of infused and AAV delivered mAb to the rectal mucosal is encouraging and the protective effect of AAV delivered antibodies against viral challenge represents an example of species matched AAV vectored immunoprophylaxis against infectious diseases.

The generation of anti-drug antibodies responses that block neutralization or binding has been observed previously in many studies attempting mAb delivery based on AAV and remains a major hurdle in the field.[27–29] In the present study, the half-life of infused antibodies did not decrease following repeated infusions, except following the fourth infusion of ITS01, by ~2-fold in one animal. In the presence of an anti-drug antibody response, this reduction would have expected to have been far more dramatic, as previously described.[39] In fact, directly assaying anti-drug antibody responses revealed levels at or close to background for all but one animal. The one animal, A03684, that showed signal just above baseline may have been falsely positive. The serum reacted to both antibodies, despite administration of only ITS09.01-LS. Additionally, no boosting of the ADA was observed after successive administrations, despite a signal just above background following the second infusion.

We achieved long term anti-SIV Env mAb expression in NHP following AAV injection. Importantly, the consistency with which these mAbs are expressed over the long term is encouraging, with expression observed for up to 1 year. Initial studies delivering immunoadhesins, antibody-like molecules derived from SIV infected rhesus macaques, demonstrated stable expression beyond one year post AAV in all animals injected.[26,30] Subsequent studies delivered whole IgG or antibody based molecules to provide anti-envelope immunity to NHP, resulting in stable transduction of a subset of those animals.[27–29] Transgene expression in these later studies suffered from anti-drug antibody generation elicited against non-rhesus epitopes (human or artificial constructs), with one group requiring immunosuppression to maintain expression beyond eight weeks. These NHP studies reveal the importance of immunological tolerance to transgene products. Rejection by anti-drug antibody has not been reported in other gene therapy clinical trials correcting hemophilia or enzyme deficiencies but remains a major hurdle for antibody delivery.[12,14,21–24,40,41]

In spite of previous reports finding strong ADA responses in 50–100% of AAV injected animals, we found only 20% of animals generating these responses against ITS01 and ITS06.02.[27–29] We confirm the loss of expression, as observed by others, following the generation of an ADA response. This reduced frequency of ADA responses is likely due to the use of autologous, naturally-arising mAbs, with high identity to germline, 91% and 88% for ITS01 and ITS06.02, respectively. The ADA responses were directed against Fabs, and were not cross reactive, ruling out anti-CH_2/3_ responses, while suggesting no inherent adjuvanting of self epitopes by AAV. The remaining possible epitopes targeted by ADA responses are thus CH1, light chain constant regions, variable region alleles of the transduced antibodies not harbored by the transduced monkey, or anti-idiotype directed against the recombined/somatically hypermutated region of the variable regions.

It is noteworthy that in our study, there was universal concordance between the loss of expression in the plasma and the generation of ADA—a strikingly contemporaneous effect. This suggests that the mechanism of loss of transgene expression in AAV-directed expression of autologous mAbs is likely because of antibody-based mechanisms, and not cellular (cytotoxic). This observation further reinforces the importance of circumventing ADA responses, as previously suggested.[27–29]

We tested various regimens to effect higher expression. Surprisingly, there was little impact of dose on expression of the transgene in the plasma, suggesting that physical limits are reached at about 10^12^ gc per injection. Notably, physical limitations of uptake by muscle cells are unlikely to be different between NHP and humans. Our data gives pause to clinically dosing AAV single-site injections based on the weight of the recipient. In contrast, we found that the expression did increase with the number of injections sites, using the same cumulative dose, as has been reported previously in the case of DNA vaccination.[42] Thus, clinical trials attempting to reach the plasma levels of mAb we found in NHP may need to scale the *number* of injections by weight—e.g., a single injection of 2 x 10^12^ gc in a 4 kg NHP is a model for 20 injections of 2 x 10^12^ gc in an 80 kg human, but will not model a single injection of 40 x 10^12^ gc.

Prior preclinical doses used for gene transfer via AAV have ranged from 3 x 10^11^–10^14^ gc/animal in macaques.[26–30] Human clinical trials deliver comparable doses at 1.6 x 1013–4 x 10^14^ gc/subject based on a 80 kg subject.[12,14,22,23,40,43] Stroes et al. administered 8 x 10^12^ and 2.4 x 10^13^ gc/subject (based on 80 kg/subject) of AAV1 to deliver a lipoprotein lipase in 40 or 60 injections respectively.[43] These authors demonstrated persistent serum lipase activity up to 31 months, supporting the proposed dose spreading. Conversely, Manno et al. administered hF.IX via AAV2 at 1.4 x 10^13^–1.3 x 10^14^ gc/subject in 10–20, 30–50 or 80–90 shots, but found low serum transgene accumulation.[40]

Our data reveal the necessity to factor anti-vector immunity into AAV based passive immunization strategies, yet another hurdle for this technology. Given the successful transduction of animals harboring low/intermediate pre-existing anti-vector immunity, the pre-existing humoral response appears to have been qualitatively different from that generated via delivery of AAV8 IM in that it did not abrogate transduction. However, the humoral response after AAV immunization is substantial, as previously reported.[14,21,23,25,40] Likely, the ineffective re-administration relates to the strong anti-AAV humoral response (which is itself boosted following the second injection). These data highlight the challenge of crafting immunization regimens that might re-administer many shots of AAV within a <1 week window. Work is underway to identify AAV capsids with low seroprevalence or immunogencity that might circumvent the issue of anti-vector immunity.[44–47]

Glycan profiles of AAV expressed mAbs demonstrate the differences between glycosylation machinery of muscle, 293, CHO, and B/plasma cells. It is widely recognized that the N267 glycan on the Fc region relates to the neutralizing, functional or inflammatory nature of a given antibody by modulating Fc receptor binding affinity.[48–52] AAV expressed mAbs bear profiles with higher frequency of digalactosylated and sialylated glycan species on their Fc versus antibodies derived via cell culture or infection. This signature has been reported to confer increased Fc mediated functionality to mAbs and lower anti-inflammatory signaling.[53] Cell line and infection derived mAbs have an opposite Fc glycan profile in this regard, based on their frequent agalactosylation and lower frequency sialylation. The diverse profile of discrete glycan species found on antibody Fc derived from various cellular sources further highlights the potential heterogeneity of antibodies produced for/following passive immunization. More work detailing specific Fc-glycan modulated effector function is needed before passive immunization approaches against target diseases can be tuned at the glycan level.

To prolong the half-life of transgene mAbs, the Fc region could be modified to augment circulating mAb levels by reducing clearance. Based on clinical data comparing WT versus “LS mutant” Fc regions, the half-life of an antibody can be prolonged by a factor of 3-fold.[36,37] We expect that introducing this mutation into the transgene Fc could therefore result in a commensurate 3-fold increase in the serum mAb levels.

Our experience highlights the importance of staged testing in mice, before proceeding to large mammals. Some constructs did not express as rapidly as others or retain neutralizing function when expressed in mice. The reasons for this discrepant in vitro neutralization are unclear, but could be related to inappropriate V region glycans (where potential N-linked glycosylation sites are present) or other post-translational modification. Screening multiple constructs in mice, then down-selecting for trials in NHP to confirm expression and function is key. The NHP model may be useful for optimizing vector parameters such as capsid (tissue targeting), the promoter/enhancer, and other molecular considerations to maximize expression. This technology may inform clinical trial design, however consistent translation between simian and human systems will need experimental validation.

Lastly, we demonstrate the ability of an AAV based VIP strategy to provide a protective effect against infectious disease. To date antibody mediated protection based on mAb delivery via AAV has only been demonstrated in clonal SHIV/SIV challenge models in macaques and HIV, Influenza, and Ebola clonal challenge in mice.[26–29,38,54,55] Here we use a fully rhesus system to delivery native mAbs and a repeated limiting dose SIVsmE660 swarm challenge model to demonstrate antibody mediated protection. Previous attempts to delivery whole IgG via AAV for protection studies have been hampered by the generation of ADA responses that abrogated transgene accumulation during the challenge regimen. Here, only one animal in our protection study had plasma mAb levels drop to below detection limits by the final challenge timepoint. We acknowledge the caveat of reliance on historic controls for the initial protection study. However, the rates of infection historic (0.33 infections/challenge) and contemporaneous controls (0.40 infections/challenge) were reproducible. These data demonstrate the utility of vectored approaches in macaques to model passive immunization against infectious disease that maintain functional levels of mAb throughout a prolonged challenge regimen. Studies to further detect and dissect an antibody-mediated protective effect are currently underway.

AAV-vectored mAbs, by virtue of retaining function, mucosal translocation, and stable expression levels, could provide a means of passive immunization against infectious diseases. This represents an attractive alternative to regimens employing repeated infusions, which may not scale well given the practical and financial costs of widespread application needed in the battle against HIV or other epidemics.[56] Clinical trials delivering broadly neutralizing antibodies against HIV have begun using either protein infusion (NCT02568215, NCT02716675) in Phase II or AAV VIP in Phase I (NCT017455, NCT03374202) and will serve as a proof of principle on these concepts. The infrastructure to scale passive immunization strategies to combat epidemic disease is not yet in place. This effort will require substantial investment from government and industry to make passive immunization either via AAV or infusion feasible on a global scale. In the past 8 years, a large number of potent broadly neutralizing antibodies have been described that target various sites of vulnerability on its viral envelope.[57–61] The best candidates for prophylaxis or therapy remain to be determined. The NHP model, as we demonstrate here, provides an excellent platform with which to compare mAbs via infusion or AAV to test and optimize passive vaccine concepts.

## Materials and methods

### Ethics statement

All animal experiments were reviewed and approved by the Animal Care and Use Committee of the Vaccine Research Center, National Institutes of Allergy and Infectious Diseases (NIAID), National Institutes of Health (NIH) and all animals were housed and cared for in accordance with local state, federal and institute policies in an American Association for Accreditation of Laboratory Animal Care-accredited facility with stringent standard operating procedures and compliant with U.S. Animal Welfare Act (AWA) and Regulations, the Public Health Service (PHS) Policy on Humane Care and Use of Laboratory Animals, the Guide for the Care and Use of Laboratory Animals and all applicable NIH Policies on *in vivo* research. These experiments were carried out under the following protocol numbers (VRC-14-475, VRC-14-499, VRC-14-487). All animals were monitored carefully by veterinary staff. To date, no adverse events have been associated with these immunizations. The NHP study was conducted in accordance with the requirements of the Animal Welfare Act (AWA) set forth under the Regulations and Standards in the Code of Federal Regulations (CFR), Title 9 CFR, Chapter 1, Subchapter A—Animal Welfare, Parts 1, 2, and 3. Approval of this study by the VRC ACUC ensured compliance with AWA requirements for environment enhancement adequate to promote the psychological and physical well-being of nonhuman primates. Specific humane interventions are described in the nonhuman primate protocol, including detailed descriptions of monitoring frequency, use of anesthesia and analgesia for procedures, and humane method of euthanasia based upon the American Veterinary Medical Association (AVMA) Guidelines on Euthanasia, or as recommended by the veterinary staff. Sedation was performed using Ketamine Hydrochloride 5.0–10.0 mg/kg IM or with xylazine 0.5–1.0 mg/kg, as required. Upon completion of the study animals were made available to other investigators according to the Vaccine Research Center Policy on post-study disposition of nonhuman primates. Any nonhuman primates that could be used in other research protocols, by criteria defined in this policy, were euthanized at the discretion of the PI via the following method: exsanguination while under a deep surgical plane of anesthesia induced by sodium pentobarbital, 10–15 mg/kg IV. Euthanasia was performed under the guidance of the veterinary staff. Euthanasia was followed by a full gross and histopathologic examination of a full range of tissues. All carcasses were disposed of by incineration.

### Study design

Our goal was to determine the feasibility of antibody gene transfer using AAV8 in rhesus macaques as a model for passive immunization interventions in humans. Healthy male and female Macaca mullatta animals were used, all were of Indian origin except where noted. Based on the literature, we expected expression of antibody might be difficult to achieve and thus began with a pilot study of 6 NHP at high dose. Given the robust expression observed, we sought to further optimize and characterize our system of passive immunization by altering dose (Indian origin macaques) and number of shots (Chinese origin macaques). Given the success of delivery at 2 x 10^12^ gc/animal, we delivered antibody via AAV8 at this dose and 5 x 10^12^ gc/animal in larger challenge studies, including relevant antibody delivery data here. Two outliers were excluded from averages of serum antibody; a control monkey with high background serum reactivity in our ITS01 quantitation and one monkey given AAV at low dose that did not show transgene expression. All macaques included our protection analysis were of Indian origin. Rates of infection and viral loads between historic and contemporaneous controls were comparable, as previously observed with this challenge stock. Two monkeys were censored after the second challenge in our initial protection study due to false positive SIV viral load measurement. Sample collection endpoints were at least 25 weeks post AAV.

### Serum antibody quantitation (MSD)

Antibody titers were quantified from heat-inactivated sera using the Meso-Scale Discovery Sector 2400 platform as previously described (Meso-Scale Discovery; Rockville, MD., USA).[62] Breifly, MSD 384 well streptavidin coated plates were blocked for 1 hr at RT using 35μl 5% (W/V) MSD blocker A buffer shaking at 600RPM. Plates were washed 6 times and incubated with a biotin-bait conjugate for 60’ at RT shaking at 600RPM. For ITS01 and ITS06.02, anti-idiotype antibodies were generated in mice as described previously (Genscript; Piscataway, NJ., USA).[63] The clones 17B4-IgG1 (mouse anti-ITS01), 8A4-IgG1 (mouse anti-ITS06.02) and 44A11-IgG1 (mouse anti-ITS52) were selected based on maximum binding to target with minimal off target reactivity. These antibodies were biotinylated and used as coat protein at 1 μg/ml. For total IgG quantitation, coat protein was mouse anti-rhesus IgG-biotin (Life Diagnostics; Westchester, PA., USA, clone 10C2-9) at 4 μg/ml. For total anti-Env antibody, SIVmac239 gp140-FT was enzymatically biotinylated using BirA according to manufacturer’s instructions (Avidity L.L.C.; Aurora, CO, USA). ITS52 in sera could not be detected and animals receiving only this antibody were excluded from all analyses, those receiving ITS01 + ITS06.02 + ITS52 were grouped with ITS01 + ITS06.02. Bait proteins were removed by washing and plates were incubated as above with sample sera diluted at 1:500, 1:2500, 1:12500, and 1:62500, purified mAb standards or blanks in 1% (W/V) MSD blocker A buffer. Samples were washed and plates were incubated as above with secondary antibody sulfo-tagged mouse anti-rhesus IgG (Southern Biotech; Birmingham, AL, USA, clone: SB108a). Plates were washed as before, followed by addition of 35μl μl of MSD read buffer. Electrochemiluminescence was quantified on an MSD sector 2400 instrument according to manufaturers recommendations. Antibody concentration in sera/mucosal extracts were calculated based on standard curve. For a given sample, the estimate of the lowest in-range dilution was included as the final estimate of mAb concentration.

### Serum antibody quantitation (ELISA)

Alternately, purified antibodies delivered during NHP PK infusion and initial mouse AAV studies were quantified in animal sera by ELISA as previously.[36] Breifly, 96-well maxisorp plates (Thermo Fisher Scientific; Waltham, MA., USA) were coated with 2 μg/ml of SIVmac239-gp140-FT in PBS at 4°C for 16 hours. Standards, blanks or diluted samples (1:50,000, 1:100,000 and 1:150,000) were applied to wells and incubated for 60’ at 37°C. Secondary anti-rhesus IgG-HRP (Southern Biotech; clone: SB108a) was diluted 1:5000 and incubated in wells for 60’ at 37°C. HRP activity was resolved using 100 μl of SureBlue TMB (KPL Inc., Gaithersburg, MD., USA) and stopped using 100 μl of 1N H_2_SO_4_. Plates were washed with PBS + 0.02% Tween-20 six times between coating, samples, detection and resolution. OD_450nm_ was measured on a Versamax microplate reader (Molecular Devices; Sunnyvale, CA., USA). Antibody concentration in sera/mucosal extracts were calculated based on standard curve. For a given sample, the estimate of the lowest in-range dilution was included as the final estimate of mAb concentration.

### Anti-idiotype antibody generation

Balb/c mice were immunized with Complete Freund’s Adjuvant (CFA) with Fab derived from ITS01 or ITS06.02 initially. At weeks 2 and 5 post immunization (p.i.), mice were reimmunized with Fab plus Incomplete Freund’s Adjuvant (IFA). At week 8 p.i., mice were reimmunized with Fab alone. Splenocytes were fused with myeloma cells at day 60 p.i. and plated in 96-well plates for expansion for screening of supernatant reactivity to Fab. Positive clones were expanded, subcloned by limiting dilution and reexpanded to generate anti-idiotype antibody producing cell lines. Culture supernants of subclones were screened to ensure Fab reactivity was preserved. Hybridoma heavy and light chain V regions sequences were determined by RT-PCR and Sanger sequencing. These VH and VK sequences were synthesized and cloned into mouse IgG1 and IgK expression vectors for production in cell culture.

### Bioconjugation

Antibodies were biotinylated (Sigma-Aldrich; St. Louis, MO., USA) or sulfo-tagged (Meso-Scale Discovery) using activated NHS-ester chemistry. Antibodies were buffer exchanged into 100 mM carbonate buffer pH 8.4 and adjusted to 1 mg/ml. NHS activated tags (biotin or sulfotag) were reconstituted in DMSO anhydrous to 10 mg/ml and 3 nM, respectively and 8 μg or 500 nmoles were added to buffer exchanged IgG. The reaction was incubated for 1 hour at RT in the dark with gentle agitation. The reactions were simultaneously desalted and buffer exchanged twice using zeba spin columns 40 MWCO (Thermo Fisher Scientific; Waltham, MA., USA) into 10 mM TRIS, 150 mM NaCl + 0.1% NaN_3_ pH 8.2.

### Mucosal antibody quantitation

Transgene antibodies were extracted from tissue biopsies and mucosal swabs as previously described.[64] Briefly, tissue biopsies were transferred to and dissociated in microtubes using a micropestle (VWR International; Radnor, PA., USA) in PBS plus protease inhibitor (Roche; Basel, Switzerland). Samples were spun at 15000 rpm at 4°C for 15 min. Supernatant was filtered using Spin-X tubes (Corning Life Sciences; Corning, NY., USA) with .22 μm sterile filter (Sigma-Aldrich) and spun as before. Antibody was extracted from muscosal swabs by soaking Wek-Cel sponge spears in 300 μl elution buffer (PBS + protease inhibitor + 0.5% IGEPAL + 0.25% BSA) for 5 minutes in Spin-X tubes with .22 μm sterile filters. Tubes containing sponges and buffer were spun as before. Following filtration, an additional 300 μl was applied to sponges and tubes were spun as before. Saliva was collected by spinning Salivette tubes (Sarstedt; Nümbrecht, Germany) at 3000 x g for 10’. Antibody in extracted samples were quantified as above using a single 1:5 dilution point for transgene antibody and 1:100–1000 for total IgG detection. Antibody concentration in sera/mucosal extracts were calculated based on standard curve. For a given sample, the estimate of the lowest in-range dilution was included as the final estimate of mAb concentration. Mucosal extracts with blood contamination and were excluded based on visual observation.

### Pseudovirus neutralization assay

The pseudovirus neutralization assay was performed as previously described.[65] In brief, mAb/serum were serially diluted 5-fold for a series of 8 dilutions and incubated with previously titrated pseudovirus for 30’ in 96-well black culture plates (Thermo Fisher Scientific). Following incubation, 10^4^ TZM-bl cells are added to wells in 20 μl and incubated overnight. The following day 80 μl additional medium was added. 24 hours later, 50 μl of Steady-Glo lysis/luciferase substrate reagent solution (Promega; Madison, WI., USA) was added to each well. The data was acquired using a Spectramax luminometer (Molecular Devices). Neutralization is calculated as a 1 –sample RLU / baseline control RLU after background subtraction.

### Pseudovirus production and titration

Pseudovirus stocks were generated and titrated. Breifly, 293T cells cultured in DMEM + 10% FBS (Thermo Fisher Scientific) were plated at 2 x 10^6^ cells/flask in a T75 flask. Cells were co-transfected on day 1 with 10 μg of ΔEnv-SG3 HIV plasmid backbone and 3.3 μg envelope expression plasmid of the desired pseudotype using 40 μl Fugene transfection reagent (Promega) in 800 μl serum free media. Fresh culture media was replaced on day 1 post transfection. Supernatant containing virus was harvested on day 2 post-transfection, filtered at .45 μ (Merck-Milipore; Billerica, MA., USA), aliquoted and frozen at—80°C.

### Antibody purification

Plasmids containing heavy and light chain genes for a given antibody were co-transfected into Expi293 cells (Thermo Fisher Scientific) at 0.5 μg/Ig chain/ml of culture containing 2.8 x 10^6^ cells/ml in 85% of the final culture volume on day 0. Expifectamine (Thermo Fisher Scientific) and DNA were diluted in optiMEM (Thermo Fisher Scientific) at 10% of the final culture volume according to manufacturer’s specifications and added to cells. On day 1, the included enhancers 1 and 2 were added to the culture according to the manufacturers specifications. Culture supernatants were harvested on days 5–8 and filtered using a .45 μ filter. Antibodies were purified from supernatant over a protein A column (GE Healthcare; Silver Spring, MD., USA) by gravity flow and buffer exchanged into PBS. All preparations were sterile filtered. Antibody preparations were stored at 4°C or frozen at -30°C.

### AAV8 production

Plasmids containing packaging component for AAV8 capsid plus the desired antibody expression transgene containing chimeric genome were co-transfected into 293 cells. Supernatants were harvested and viral vectors were precipitated using PEG8000 (Sigma-Aldrich) and pelleted by centrifugation. Viral pellets were subjected to 2 rounds of CsCl centrifugation (60,000 RPM x 24 hours) and AAV factions were collected via syringe. Vectors were desalted and concentrated to 3–3.6 x 10^13^ gc/ml and frozen. All AAV lots were tested for purity via SDS-PAGE and for expression in SCID mice.

### AAV immunization

To screen constructs, SCID mice were immunized with 2.5x10^10^ gc total of AAV8-DJ vectors (n = 1 per construct) containing anti-SIV Env antibody genes via the intramuscular injection into the gastrocnemius in a volume of 100μl. Non-human primates were immunized with AAV8-DJ vectors containing anti-SIV Env antibody genes via the intramuscular route. Intramuscular injection was distributed over 1, 2, 4, or 8 injections into the right quadriceps, both quadriceps, both quads and gastrocnemius, or quads, gastrocs, biceps brachii and deltoids (respectively). Injection volumes were 250 or 500 μl for a total dose between 2 x 10^11^ and 2 x 10^13^ gc/animal.

### Anti-AAV immunity

Anti-vector immunity was determined via ELISA as described above. Coat protein was empty AAV8 particles at 2 x 10^10^ gc/ml in PBS. Serum samples were serially diluted 5-fold over 8 dilutions and applied to plates following coating and blocking. Anti-Rhesus IgG (Southern Biotech; clone SB108a) was used for detection. Plates were resolved and readings acquired as above.

### Antibody infusions

Purified antibodies were infused into rhesus macaques at 10 or 30 mg/kg intravenously four times at approximately nine week intervals into the saphenous vein using a 20–22 g needle and syringe (Becton, Dickinson and Co.; Franklin Lakes, NJ, USA). Blood draws were sampled from the saphenous vein on the opposite leg.

### Anti-drug antibody quantitation

Anti-drug antibody responses were detected using MSD sector 2400 as above using biotinylated ITS01-Fab or ITS06.02-Fab as coat protein at 0.5 and 2 μg/ml, respectively. Fabs were generated by digesting 1mg whole IgG containing a cleavage site (HRV3C) from human rhinovirus by incubation at 4°C for 16 hours with 10 U of HRV3C protease (Thermo Fisher Scientific). Fabs and Fc were separated by protein A affinity chromatography (GE Healthcare) and further purified by size exclusion chromatography using an ÄKTA FPLC (GE Healthcare). 17B4 and 8A4 expressed on rhesus IgG1 Fc were used as standards to provide an estimate of serum anti-drug antibody. Serum ADA and standards were detected using an Fc reactive anti-human IgG at 0.25 μg/ml. ADA responses against ITS09.01-LS were measured via as above, however, no positive control anti-ITS09.01-LS clone was available on a rhesus IgG1 Fc backbone and relative units are electrochemiluminescence units.

### Glycosylation analysis

Magnetic streptavidin beads (New England Biolabs; Ipswich, MA., USA) were linked to SIVmac239-biotin according to manufacturers instructions at a ratio of 1 μg protein/10 μl beads and washed with binding buffer (0.5 M NaCl, 20 mM Tris-HCl (pH 7.5), 1 mM EDTA) prior to use. 200 μl of heat-inactivated plasma was incubated with 50 μl of 1 μm blank beads for 1 hour at RT to clear out any bead-reactive antibodies. Beads were precipitated, and plasma was added to 25 μl of antigen-bound magnetic beads at 37°C for 1 hour. Beads were then washed with binding buffer and resuspended in 20 μl of PBS containing of IdeZ Protease (New England Biolabs) and incubated at 37°C for 1 hour while shaking to release IgG F_c_ fragments from the beads. Cleaved F_c_ fragments were deglycosylated and labeled using the GlycanAssure APTS kit (Thermo Fisher) according to manufacturer’s instructions. Briefly, glycans were removed using PNGaseF at 50°C for 1 hour. Glycans were purified on magnetic beads prior to being labeled with APTS for 2 hours at 50°C. Excess dye was removed using glycan-reactive magnetic beads. Resultant labeled glycans were diluted 1:5 in a total volume of 10 μl of capillary electrophoresis loading buffer containing GeneScan 600 LIZ Dye Size Standard v2.0 and Landmark Red and run on a 3730 Genetic analyzer using a POP7 polymer and a 36 cm capillary according to manufacturers instructions. Data was analyzed using the GlycanAssure Data Analysis Software (Thermo Fisher) against 24 labeled standards (Prozyme; Hayward, CA., USA).

### Blood withdrawals

The primary site for biosampling was the femoral vein but other sites such as the saphenous or cephalic tail veins were used according to the approved protocol, at the discretion of veterinary staff. Bleeds were 5 ml in serum separator tubes (SST) or EDTA tubes using 20–22 g needles and either syringes or vacuum tubes (Becton Dickinson and Co.). The blood volumes removed from each monkey were based on: 1) the current assessment that total blood volume of a rhesus monkey is approximately 6% of its total body weight (60 g/kg); 2) the maximum blood volume removed did not exceed 20% (12 ml/kg) per month, with no more than 15% (9 ml/kg) removed during any single draw; and 3) ongoing experience and hematological observations in each monkey.

### Intrarectal challenge

Intrarectal challenges were performed according to the current SOP from our laboratory of animal medicine, LAM SOP 9008–3. Briefly, challenge stocks were titrated in vivo as described previously[66]. On the day of challenge, viral stock aliquots were thawed at RT and diluted in PBS to the appropriate dilution to make 1ml aliquots in 1ml syringes. Animals were sedated with Ketamine (10 mg/kg body weight (BW), intramuscularly (I/M) or subcutaneously (S/Q)) and Xylazine (0.5 mg/kg BW, I/M or S/Q) on the day of challenge and laid prone with elevated haunches. Syringes were inserted intrarectally up to the end of the barrel and the viral challenge stock was injected. Syringes were removed after 5 minutes and animals were recaged.

### Viral load measurement

Plasma SIV gag RNA was quantified to determine viral loads as previously described.[67]

### Statistics

All statistical analyses were performed using JMP statistical analysis software (SAS Institute Inc.; Cary, NC, USA). Differences in antibody expression levels over time were calculated using a matched pairs model to perform a Wilcoxon signed rank test. Correlation of relative mucosal and serum mAb were performed by linear fit of the Log_10_ of the data by compartment (rectal, nasal, or saliva sponges, or rectal biospies) at weeks 2, 4, or 5–24. Covariates analysis was performed on Log_10_ of relative mucosal data to control for contributions of dose and number of injections in AAV immunized animals. Tests between multiple groups were performed using nonparametric Steel-Dwass to adjust for multiple comparisons. Comparisons between two groups were performed using Wilcoxon/Kruskal-Wallis Rank Sums test where data were not normally distributed. Comparison of mean serum antibody levels between transgenes across time for statistically significant differences was performed using matched pairs analysis. Smoothed fits were generated using R (version 3.4.0).

## Supporting information

S1 Fig Pharmacokinetics of infused anti-SIV env mAbs. ITS01-LS (blue; n = 2) and ITS09.01-LS (red; n = 2) were infused intravenously 4 times (indicated on x-axis) into NHP at 30 mg/kg (solid lines) or 3 mg/kg (hashed line) and measured by ELISA. The absolute serum (A) and fractional mucosal (B) expression in rectal biopsies (upper) and swabs (lower) are shown. (C) Fractional mucosal mAb correlated with fractional serum mAb for both biopsies (left) and swabs (right); linear regression is fit (solid lines) with a 95% confidence interval (shaded area). (D) Serum mAb half lives are expressed in days by animal and infusion number in table format, with average and standard deviation across all four infusions.
(EPS)

S2 Fig Lack of measurable ADA elicitation after mAb infusion. Rhesus macaques were infused with ITS01-LS (n = 2, blue) or ITS09.01-LS (n = 2, red) four times (denoted on x-axis by numbered circles) over the course of ~60 weeks. Averages of duplicate measurements of anti-drug antibody responses against ITS01-LS (upper) or ITS09.01-LS (lower) for all four animals are shown. Anti-ITS01-LS relative units are standardized based on a monoclonal anti-ITS01-LS standard; anti-ITS09.01-LS relative units are expressed in electrochemical units as no anti-idiotypic mAb has been isolated. Limits of detection are denoted by hashed lines for ITS01-LS and ITS09.01-LS.
(EPS)

S3 Fig Screening potential AAV8 constructs in mice. (A) Antibody concentration as measured by ELISA in mice at weeks 2, 4 and 8 (red, green, blue, respectively) injected with AAV8-Ab (2.5 x 10^10^ gc/animal) (ND, not done). (B) Neutralization regression curves are shown for ITS01, ITS08, ITS10, ITS11, ITS06.02 (panels, left to right) from purified mAb (blue, open circles) or sera from AAV injected mice (red, open squares) from 2 replicates at 3 timepoints, against either the SIV tier 1 smE660.A8 or the tier 2 smE660.2A5.
(EPS)

S4 Fig AAV bolus injection saturates injection site at high doses. (A) Absolute and (B) fractional serum antibody levels of ITS01, ITS06.02 or both for NHP that received 2 injections into the quadriceps with AAV8-ITS01 and/or AAV8-ITS06.02 at low, medium, high or zero doses (2 x 10^11^ (n = 2), 2 x 10^12^ (n = 24), 2 x 10^13^ (n = 6) gc/animal, respectively), compared with controls (n = 16) receiving no injections. (C) Absolute and (D) fractional serum antibody levels of ITS01, ITS06.02 or both for NHP injected with 2 x 10^12^ gc/animal in 1, 2, 4, 8 shots: into the right quadriceps (n = 2), both quadriceps (n = 24), both quadriceps and gastrocnemii (n = 2) or both quadriceps, gastrocnemii, deltoids and biceps (n = 2). Serum transgene mAb concentrations are plotted at peak (left) and set point (right), stratified by dose (E) (all animals receive 2 shots; Low = 2 x 10^11^ gc/animal, Med = 2 x 10^12^ gc/animal, High = 2 x 10^13^ gc/animal) or number of shots (f) (all animals receive 2 x 10^12^ gc total). Animal ZG57 (Low dose) has been excluded due to lack of transduction. Asterisks indicate statistical significance (p ≤ 0.05).
(EPS)

S5 Fig Delivery does not impact transport of AAV expressed mAb to mucosa. Fractional mucosal Ab translocation is shown for AAV injected NHP for nasal, rectal and buccal compartments (top, middle, bottom, respectively), stratified by dose (all receive 2 shots, left) or number of shots (all receive 2 x 10^12^ gc/animal total, right). Data points outside the linear range of standard curves were excluded. No nasal swab measurements for the low dose animal (2 x 10^11^ gc/animal) were detectable (upper left).
(EPS)

S6 Fig Saliva and nasal mAb abundance correlates with serum at late timepoints. (A) Averaged fractional mucosal mAb levels for nasal mucosa (red), rectal mucosa (green) and saliva (blue) with shaded 95% confidence interval. (B) Relative nasal (left) and buccal (right) mucosal mAb levels at weeks 2, 4 and 5–24 (top, middle, and bottom, respectively).
(EPS)

S7 Fig Passively infused in vitro produced Ab exhibits clearance of selected glycosylated Ab species. Glycans were eluted from serum anti-SIV env reactive mAbs from passively immunized animals and analyzed via capillary electrophoresis. (A-D) Grouped glycan species are displayed as a percent of total glycans for animals receiving infusion of purified NAb are compared across time. (E) Individual species of glycans are quantified as a percent of total and compared between across time for infused animals.
(EPS)

S8 Fig Anti-SIV env antibody Fc-glycan relative abundance by source. The relative abundance of Fc-glycan components ((A); Total Glycans) or species ((B); Individual Glycans) are expressed as greater than or equal to by antibody source where differences in groups were found to be statistically significant p ≤ 0.001.
(EPS)

S1 Table List of animals included in challenge analysis. (EPS)
